# Prognostic and predictive value of VHL gene alteration in renal cell carcinoma: a meta-analysis and review

**DOI:** 10.18632/oncotarget.14704

**Published:** 2017-01-17

**Authors:** Bum Jun Kim, Jung Han Kim, Hyeong Su Kim, Dae Young Zang

**Affiliations:** ^1^ Division of Hemato-Oncology, Department of Internal Medicine, Hallym University Medical Center, Hallym University College of Medicine, Seoul 07441, Republic of Korea

**Keywords:** renal cell carcinoma, von Hippel-Lindau, anti-VEGF therapy, prognosis, meta-analysis

## Abstract

The von Hippel-Lindau (VHL) gene is often inactivated in sporadic renal cell carcinoma (RCC) by mutation or promoter hypermethylation. The prognostic or predictive value of VHL gene alteration is not well established. We conducted this meta-analysis to evaluate the association between the VHL alteration and clinical outcomes in patients with RCC. We searched PUBMED, MEDLINE and EMBASE for articles including following terms in their titles, abstracts, or keywords: ‘kidney or renal’, ‘carcinoma or cancer or neoplasm or malignancy’, ‘von Hippel-Lindau or VHL’, ‘alteration or mutation or methylation’, and ‘prognostic or predictive’. There were six studies fulfilling inclusion criteria and a total of 633 patients with clear cell RCC were included in the study: 244 patients who received anti-vascular endothelial growth factor (VEGF) therapy in the predictive value analysis and 419 in the prognostic value analysis. Out of 663 patients, 410 (61.8%) had VHL alteration. The meta-analysis showed no association between the VHL gene alteration and overall response rate (relative risk = 1.47 [95% CI, 0.81-2.67], P = 0.20) or progression free survival (hazard ratio = 1.02 [95% CI, 0.72-1.44], P = 0.91) in patients with RCC who received VEGF-targeted therapy. There was also no correlation between the VHL alteration and overall survival (HR = 0.80 [95% CI, 0.56-1.14], P = 0.21). In conclusion, this meta-analysis indicates that VHL gene alteration has no prognostic or predictive value in patients with clear cell RCC.

## INTRODUCTION

Renal cell carcinoma (RCC) is the most common kidney cancer [1], and approximately 63,000 and 5,000 new cases of kidney cancer are diagnosed each year in the United States and Korea [2, 3]. Although complete surgical resection is considered to be curative treatment option at early stage, about 50% of patients experience disease recurrence after curative resection and up to 30% of patients have metastases at the time of the initial diagnosis [4].

The von Hippel-Lindau (VHL) gene is a tumor suppressor gene and VHL gene alteration occurs in 50-70% of clear cell RCC [5, 6]. VHL gene has an important role in regulation of the hypoxia pathway via the hypoxia inducible factors (HIFs) in sporadic RCC [7]. Functional loss of VHL protein which is induced from VHL gene alteration allows HIFs to act as a transcription factor of various pro-tumorigenic genes including vascular endothelial growth factor (VEGF) and subsequently leads to RCC development and progression by inducing angiogenesis [8].

Many therapeutic approaches to target this molecular pathway have been investigated in RCC patients and various VEGF-targeted agents yielded successful results [9–13]. Although VHL gene alteration plays a key role in RCC pathogenesis and provides plausible therapeutic target for anti-VEGF therapy, the clinical significance of VHL gene alteration in RCC has not been well established. We conducted this meta-analysis of previously published studies to investigate the predictive and prognostic value of VHL gene alteration in patients with RCC.

## RESULTS

### Results of search and eligible studies

**Figure 1:**
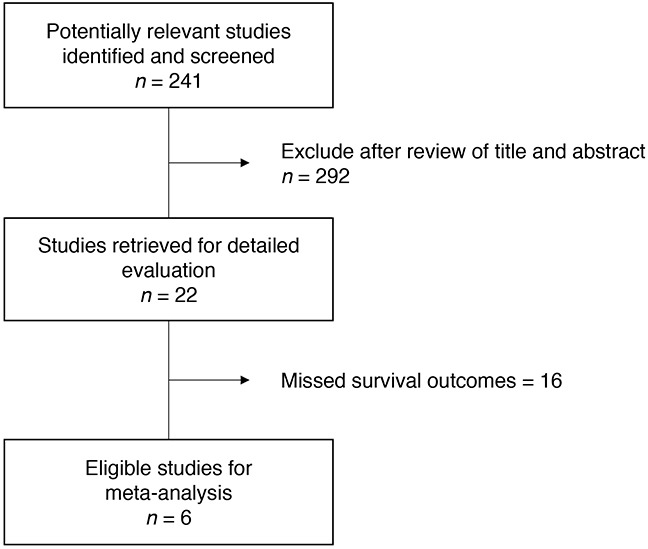
Flow diagram of searching process

Figure 1 is the flowchart of studies assessed through the searching process. Based on our searching criteria, 22 studies were retrieved for detailed evaluation. Among them, 16 studies missed survival outcomes and were excluded from the study. Finally, the remaining 6 studies [14–19] fulfilled our inclusion criteria and were included in the meta-analysis. Three studies [14–16] with 244 patients were analyzed to investigate if VHL gene alteration acts as a predictive biomarker of VEGF-targeted therapy and the other 3 studies [17–19] with 419 patients were included in the survival analysis. All patients included in the six studies had clear cell RCC.

### VHL alteration

The incidence of VHL gene alteration was various from 57% to 90% among the six studies. Out of a total of 663 patients, 410 (61.8%) had VHL alteration. Four studies [15–18] also provided the data of ‘loss of function (LOF)’ mutation and the rate of LOF mutation ranged from 38% to 58%.

### VHL gene alteration and outcomes to VEGF-targeted therapy

Table 1 lists 3 studies included in the predictive marker analysis. One [14] was a prospective randomized phase II study and the other two [15, 16] were retrospective studies. While various VEGF-targeted agents including sunitinib, sorafenib, bevacizumab, and axitinib were used in two retrospective studies [15, 16], pazopanib was used in the prospective study [14].

**Table 1:** Summary of three studies evaluating the impact of VHL alteration on the efficacy of VEGF-targeted therapy in RCC

Study	Histological subtype	Alteration analysis	N	VHL status [all mutation (LOF mutation)/hypermethylation)]		VEGF-targeted agents used	ORR (%)	RR for ORR (95% CI)	PFS (months)	HR for PFS (95% CI)
Choueiri *et al*.(2013)	ccRCC	PCR	78	VHL alteration	70 [68(NA)/8]	Pazopanib	41.4	1.18 (0.26-5.33)	17.4 (median)	0.69 (0.16-3.06)
				Wild type	8		37.5		13.8 (median)	
Choueiri *et al*. (2008)	ccRCC	PCR	123	VHL alteration	72 [60(47)/12]	Sunitinib, Sorafenib, Bevacizumab, Axitinib	49.3	1.48 (0.69-3.14)	NA	1.15 (0.76-1.74)
				Wild type	51		31.4		NA	
Rini *et al*. (2006)		PCR	43	VHL alteration	26 [25(18)/1]	Sunitinib Axitinib Interferon α + bevacizumab	48	1.69 (0.48-6.00)	10.8 (median)	0.80 (0.40-1.56)
				Wild type	17		35		5.5 (median)	

VHL, von Hippel-Lindau; LOF, loss of function; VEGF, vascular endothelial growth factor; ORR, overall response rate; RR, relative risk; PFS, progression-free survival; HR, hazard ratio; ccRCC, clear cell renal cell carcinoma; PCR, polymerase chain reaction; NA, not available

**Figure 2:**
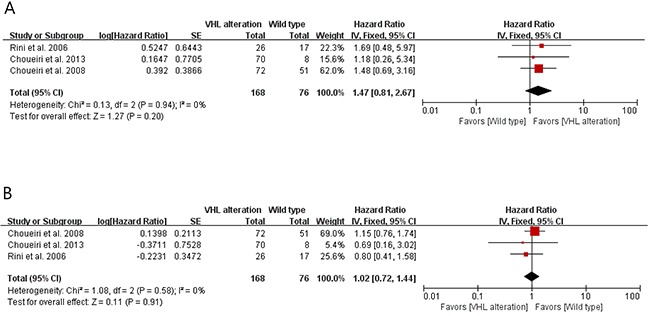
Forest plots for the association between VHL gene alteration and outcomes of VEGF-targeted therapy **A**. The relation between VHL gene alteration and overall response rate to VEGF-targeted therapy. **B**. The relation between VHL gene alteration and progression-free survival to VEGF-targeted therapy.

These studies investigated the clinical impact of VHL alteration on the efficacy of VEGF- targeted therapy in patients with metastatic RCC. When patients with VHL gene alterations compared with the wild-type patients, the pooled relative risk (RR) for overall response rate (ORR) was 1.47 [95% confidence interval (CI), 0.81-2.67], with no statistical significance (P = 0.20) (Figure 2A).

We also calculated hazard ratio (HR) and 95% CI for progression-free survival (PFS) by analyzing Kaplan-Meier curves of the studies. As shown in Figure 2B, there was no significant association between the VHL gene alteration and PFS to VEGF targeted therapy in patients with metastatic RCC (HR = 1.02 [95% CI, 0.72-1.44], P = 0.91). The results had no evidence of heterogeneity.

### VHL gene alteration and overall survival

**Figure 3:**
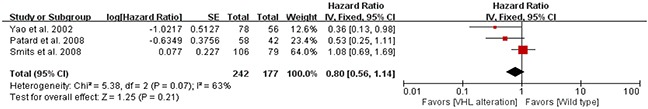
Forest plot for the association between VHL gene alteration and overall survival

Table 2 shows 3 studies included in the prognostic marker analysis. We pooled the survival data from the studies to evaluate the association of VHL alteration and overall survival (OS) in patients with metastatic RCC. Two studies [17, 18] directly presented HR and 95% CI for OS. In the remaining one study [19], we calculated HR and 95% CI by analyzing Kaplan-Meier curve. As shown in Figure 3, there was no significant association between the VHL alteration and OS (HR = 0.80 [95% CI, 0.56-1.14], P = 0.21), with no heterogeneity between the studies.

**Table 2:** Summary of three studies evaluating the association between VHL alteration and prognosis in RCC

	Histologic subtype	Alteration Analysis	N	VHL status [all mutation (LOF mutation)/hypermethylation)]		RFS (months)	HR for RFS (95% CI)	OS	HR for OS (95% CI)
Smits *et al*. (2008)	ccRCC	PCR+DS	185	VHL alteration	106 [100(97)/16]	NA	NA	NA	1.08 (0.69-1.68)
				Wild type	79	NA	NA	NA	
Patard *et al*. (2008)	ccRCC	PCR	100	VHL alteration	58 [58(58)/NA]	NA	NA	2-year RCC-SS: 84%	0.53 (0.25-1.09)
				Wild type	42	NA	NA	2-year RCC-SS: 61%	
Yao *et al*. (2002)	ccRCC	DNA-SSCP+DS	134	VHL alteration	78 [NA/NA]	138 (median)	0.40 (0.18-0.91)	144 months (median)	0.36 (0.13-0.97)
				Wild type	56	116 (median)		125 months (median)	

VHL, von Hippel-Lindau; LOF, loss of function; RFS, relapse-free survival; HR, hazard ratio; OS, overall survival; ccRCC, clear cell renal cell carcinoma; RCC-SS, renal cell cancer-specific survival; PCR, polymerase chain reaction; DS, direct sequencing; DNA-SSCP, DNA-single-strand conformation polymorphism; NA, not available

## DISCUSSION

VHL gene has an important role in regulation of the hypoxia pathway via the HIFs in sporadic RCC [7]. With functional loss of VHL protein, HIFs can act as a transcription factor of various pro-tumorigenic genes including VEGF [8]. VHL alteration also provides therapeutic target for anti-angiogenic treatment [9–13]. Therefore, it is logical to assume that VHL gene alteration might have important implications for disease prognosis. Brauch *et al*. reported that the presence of VHL mutation or hypermethylation correlated significantly with a poor risk factor (pT3 tumor grade) [20]. However, the role of VHL alteration as a prognostic factor was controversial in patients with RCC [17–19, 21, 22]. In this study, we explored if the VHL gene alteration was associated with outcomes to VEGF-targeted therapy and OS. The meta-analysis of six relevant studies showed no association between the VHL alteration and ORR or PFS to VEGF-targeted agents in patients with clear cell RCC. There was also no correlation between the VHL alteration and OS.

In this study with a total of 663 patients, 410 (61.8%) had VHL alteration. VHL gene alteration is a broad concept of genetic abnormality which includes VHL gene mutation, promoter hypermethylation, and loss of heterozygosity. These various types of VHL gene alteration can be divided into ‘LOF’ mutation which leads to truncated VHL proteins and DNA sequence variants of unknown biological consequence. Interestingly, several studies reported that the LOF mutation rather than other types of VHL alteration showed meaningful relationship with survival outcome and response to VEGF-targeted therapy in patients with RCC [15–17, 20, 21]. LOF mutation acted as a good predictive marker with better response rate and prolonged time to progression in RCC patients who received VEGF-targeted therapy [15, 16]. Other studies showed that LOF mutation of VHL gene was significantly associated with poor survival outcome [23, 24]. In this meta-analysis, the rate of LOF mutation ranged from 38% to 58%. Because VHL gene alteration was not classified into subgroups in most studies, however, we could not evaluate the role of LOF mutation as a predictive or prognostic marker

Recently, a number of studies have been conducted to identify molecular prognostic factors in metastatic RCC. The whole exome sequencing identified at least 19 significantly mutated genes in clear cell RCC, including VHL, PBRM1, SETD2, KDM5C, PTEN, BAP1, mTOR, and TP53 [25]. These mutated genes may serve as potential prognostic markers and are now under investigation. One study reported that clear cell RCC patients with BAP1 mutation showed poor prognosis, compared to those with BRM1 mutation [26]. Since the approval of VEGF-targeted agents, significant efforts have been undertaken to discover useful and clinically relevant biomarkers to predict the outcomes to anti-VEGF therapy [27–32], but no biomarkers are ready for routine clinical use. In one study examining the pharmacokinetics of sunitinib, an A-allele in CYP3A5, an absence of CAT copy in NR113 haplotype and TCG copy in ABCB1 were all predictive of improved PFS or OS and are regarded as potentially meaningful biomarkers [33].

This study has several limitations. First, the small number of studies was included in the meta-analysis and patients were treated with no identical modality. Second, this study inevitably has inherent selection bias due to the retrospective nature of included studies. Lastly, because VHL gene alteration was not classified into subgroups in most studies, we could not determine if LOF mutation has predictive or prognostic value in RCC patients.

In conclusion, this meta-analysis demonstrates that VHL gene alteration is not associated with outcomes to VEGF-targeted therapy and OS in patients with clear cell RCC. However, further studies are needed to reveal the predictive or prognostic role of certain subtypes of VHL gene alteration in patients with RCC.

## MATERIALS AND METHODS

### Searching strategy

A systematic search of PUBMED, MEDLINE, and EMBASE was performed (up to September 2016). The following searching terms were used: ‘kidney or renal’, ‘carcinoma or cancer or neoplasm or malignancy’, ‘von Hippel-Lindau or VHL’, ‘alteration or mutation or methylation’, ‘prognostic or predictive’, ‘VEGF-targeted’. All eligible studies were retrieved and their bibliographies were checked for other relevant publications.

Studies that met the following inclusion criteria were included in the meta-analysis: all patients were diagnosed with histologically confirmed RCC; data on survival or treatment outcomes to VEGF-targeted therapy in association with VHL gene alteration were available; HRs for time-dependent end-points and RR for response rates were presented or could be calculated from the data.

### Data extraction

Data extraction was done independently by two authors (BJK and JHK). If these two authors could not reach a consensus, another author was consulted to resolve the dispute. The following data were carefully extracted from all eligible studies: first author's name, year of publication, number of patients, histologic subtype of RCC, alteration status of VHL gene, VEGF-targeted agents used, ORR to anti-VEGF therapy, RR for ORR and PFS stratified by VHL alteration, and HR with 95% CI for PFS and OS.

### Statistical analysis

The association between VHL gene alteration and survival outcome was presented as HR with 95% CI. The correlation between VHL gene alteration and ORR to VEGF-targeted therapy was expressed as RR. HR, RR, and their respective 95% CI were obtained directly or indirectly from the original article. If the HR and 95% CI were not presented, we calculated them with the published methods [34, 35]. Engauge digitizer version 9.1 was used to read and analyze the Kaplan-Meier curves of the included studies. A fixed effect model was used to calculate the pooled HR estimate. HRs for death were combined using an inverse variance method based on a logarithmic conversion; 95% CI was used to determine the standard error (SE) using the formula SE=95% CI/1.96. Heterogeneity between studies was estimated using the *I*^2^ inconsistency test and chi-square-based Cochran's *Q* statistic test [36] in which *P*< 0.05 is taken to indicate the presence of significant heterogeneity. The Z-test for overall effect and its two-sided P-value were also assessed. RevMan version 5.2 software was used to report outcomes.
